# A comprehensive overview and evaluation of circular RNA detection tools

**DOI:** 10.1371/journal.pcbi.1005420

**Published:** 2017-06-08

**Authors:** Xiangxiang Zeng, Wei Lin, Maozu Guo, Quan Zou

**Affiliations:** 1 School of Information Science and Engineering, Xiamen University, Xiamen, China; 2 School of Electrical and Information Engineering, Beijing University of Civil Engineering and Architecture, Beijing, China; 3 School of Computer Science and Technology, Tianjin University, Tianjin, China; University of Canterbury, NEW ZEALAND

## Abstract

Circular RNA (circRNA) is mainly generated by the splice donor of a downstream exon joining to an upstream splice acceptor, a phenomenon known as backsplicing. It has been reported that circRNA can function as microRNA (miRNA) sponges, transcriptional regulators, or potential biomarkers. The availability of massive non-polyadenylated transcriptomes data has facilitated the genome-wide identification of thousands of circRNAs. Several circRNA detection tools or pipelines have recently been developed, and it is essential to provide useful guidelines on these pipelines for users, including a comprehensive and unbiased comparison. Here, we provide an improved and easy-to-use circRNA read simulator that can produce mimicking backsplicing reads supporting circRNAs deposited in CircBase. Moreover, we compared the performance of 11 circRNA detection tools on both simulated and real datasets. We assessed their performance regarding metrics such as precision, sensitivity, F1 score, and Area under Curve. It is concluded that no single method dominated on all of these metrics. Among all of the state-of-the-art tools, CIRI, CIRCexplorer, and KNIFE, which achieved better balanced performance between their precision and sensitivity, compared favorably to the other methods.

## Introduction

Circular RNA (circRNA) is a class of noncoding RNA that was discovered decades ago [1]; however, its abundance and ubiquity in eukaryotes were only recognized recently [2–5] because of the advance of next-generation RNA sequencing (RNA-Seq). Although it appears that many circRNAs remain to be discovered, ongoing studies continue to demonstrate important functions of circRNA in cell physiology [6–11]. For instance, the well-known circular RNA sponge for *miR-7* (*ciRS-7*), which originates from the vertebrate cerebellar degeneration-related 1 (*CDR1*) antisense transcript, has the capacity to serve as a microRNA (miRNA) sponge. It is highly expressed in human and mouse brain cells [2, 12]. Given its possession of more than 60 *miR-7* binding sites [2, 13], it is suggested to inhibit the binding of *miR-7* to its target mRNAs. Another circRNA that can potentially also act as an miRNA sponge is derived from the murine sex-determining region Y (*Sry*) gene; it is a testis-specific circRNA, possessing 16 target sites for *miR-138* in mouse [13]. Another function proposed for circRNAs is that they affect gene regulation by competing with linear splicing on the usage of splice sites during the cotranscription process, leading to a change in the level of gene expression [14, 15]. Although most circRNAs are from exons, there are also intron-containing circRNAs. It’s reported that they are largely concentrated in the nucleus [16, 17]. Intriguingly, evidence suggests that these intron-containing circRNAs enable the regulation of gene transcription in cis. Specifically, they can enhance the RNA polymerase II (Pol II) transcription activity of their parental genes, although the underlying mechanism is still not fully understood [16, 17]. CircRNAs are characterized by their noncollinearity, in which a splice donor attacks an upstream acceptor, forming a covalently closed circular structure [1, 2, 4, 12, 18–20]. This characteristic endows them with the ability to escape exonuclease digestion, enabling them to persist longer in the cell than their linear counterparts [3, 21, 22]. This feature in combination with their ubiquity in cancer tissues [23, 24], saliva [25, 26], blood [27–29], and exosomes [30, 31] suggests that circRNAs are promising as biomarkers for diseases. On the other hand, their circular structure also serves as a key element for the detection of circRNAs. Specifically, the backsplicing junction reads within the structure presented in RNA-Seq data facilitate the genome-wide identification of this RNA species, although other mechanisms such as genomic tandem duplication, template switching during PCR amplification, or trans-splicing between precursor mRNAs (pre-mRNAs) can also potentially generate such reads [7, 19] and complicate the detection process. To resolve this issue, Jeck et al. [3] developed a biochemical strategy termed CircleSeq that involves treating samples with an exonuclease that digests linear RNAs but preserves circRNAs (RNase R). However, it has been asserted that RNase R resistance alone cannot be used to determine whether an isoform is circular or not, because some circRNAs were found to be susceptible to this exonuclease [3, 19, 32, 33]. CircRNA expression is reported to be specific to different tissues/cell lines and developmental stages [32–35]. Despite the fact that some circRNAs have been experimentally verified to be abundantly expressed, even more highly than their linear counterparts, the vast majority of them are usually expressed at low levels [3, 32, 36]. This not only constitutes another challenge for their identification, but also raises doubts about their functions, indicating that the majority of them may be inert byproducts of noncanonical pre-mRNA splicing [3, 32].

The advent of high-throughput next-generation sequencing technology has enabled the sequencing of hundreds of millions of short reads, and its single-base-pair resolution provides a precise and efficient way to identify circRNAs. The detection of circRNAs from RNA-Seq data can be achieved using various software packages. There are approximately a total of 11 different tools that have been developed for this purpose. However, despite the development of this range of computational tools, no systematic evaluations of their performance have been performed. Although some attempts have been made to compare several of these packages [7, 37] and some comparisons were included in papers by those who developed these tools [33, 38–42], different conclusions were drawn with regard to their performance, owing to different subsets of tools being compared, different filtering strategies being applied, or diverse datasets being utilized, among others. The fact that some circRNAs are susceptible to exonucleases and most of them are expressed at low levels means that there is an inherent bias in filtering for reliable circRNA candidates based on resistance to RNase R and/or the selection of backsplicing junction reads above a specific abundance threshold [33, 43]. For example, recently, Hansen et al. found that, when focusing on the top 100 most highly expressed candidates detected by one tool alone, a large number (77%–88%) of the candidates predicted by 3 of the 5 tools evaluated would be qualified as artefacts, based on the criteria of RNase R resistance [37].

In this study, we perform a comprehensive evaluation of 11 different circRNA detection tools, with the aim of providing useful guidelines for researchers engaged in this field. These tools have been run and compared on 4 different datasets: (1) positive dataset: a dataset of simulated reads, encompassing a total of 14,689 circRNAs detected in HeLa cells from CircBase [44]; (2) background dataset: a large negative dataset comprised of reads generated from mRNA sequences deposited in the NCBI Reference Sequence (RefSeq) database; (3) mixed dataset, generated by combining the positive and background datasets together; and (4) real datasets. These real datasets were established by downloading 6 runs of rRNA-depleted RNA-Seq data from NCBI Sequence Read Archive (SRA), including 4 runs of RNA-Seq data from the HeLa cell line and 2 runs from an immortalized human fibroblast cell line (Hs68), of which, 2 runs of RNA-Seq data from the HeLa cell line and 1 run from Hs68 were further treated with RNase R enzyme during sample preparation. The performance of the software packages was evaluated based on metrics such as sensitivity, precision, F1 measure, Area under Precision-Recall Curve (AUC), memory (i.e., Random Access Memory [RAM]) consumption, running time, and physical disk space utilized. Notably, a striking difference in the use of physical disk space was observed among these tools, which is an important factor that should not be overlooked when running software on a large dataset or several moderately sized datasets in parallel.

## Results

### Evaluation with the positive dataset

**Fig 1:**
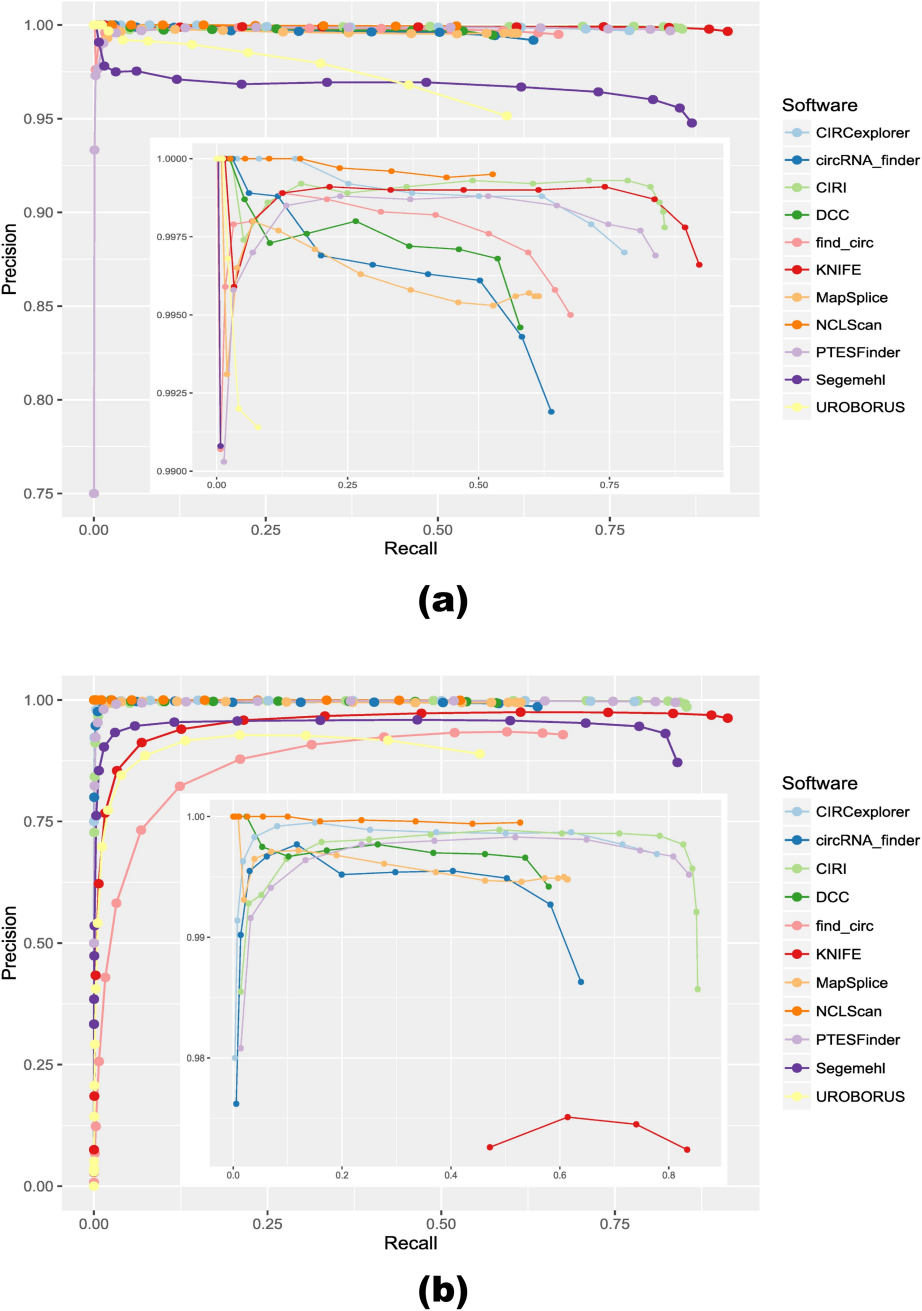
Precision-recall Curve for the 11 circular RNA detection tools on synthetic positive and mixed datasets. Counts of candidates and true positives were calculated at increasing thresholds for the number of supporting reads, and the precision-recall results for each tool were further computed and depicted in the figures above. **(a)** Positive dataset (Inset: precision above 0.99 was detailed). **(b)** Mixed dataset (Inset: precision above 0.97 was detailed).

As stated above, the positive dataset comprises 14,689 circRNAs derived from those detected in HeLa cells deposited in CircBase, with the number of supporting read pairs ranging from 2 to 24 and circle size varying from 51 to 846,530 base pairs (bps). We applied the 11 circRNA detection tools to identify circRNAs on this dataset. Table 1 show that most tools achieved high precision (>94%) and varying sensitivity (52%–93%). As F1 score (i.e., F1 = (2 * precision * sensitivity)/(precision + sensitivity)) weights precision and sensitivity equally and serves as a good metric to indicate whether a tool achieves favorable precision and sensitivity simultaneously, the performance of each method in terms of F1 was also included in Table 1. In summary, regarding the F1 measure, KNIFE, CIRI, PTESFinder, Segemehl, and CIRCexplorer were the top 5 performers on this dataset, with an F1 score above 0.85. Moreover, the effect of filtering for reliable circRNAs by increasing supporting read counts is shown in Fig 1A. In general, we observed that the precision of each tool increased with thresholds for read counts, but some highly expressed false positives were also reported by several tools, leading to a fluctuation of precision at the end. Also, we could observe that NCLScan consistently dominated other tools regarding the precision measure. Meanwhile, KNIFE, Segemehl, CIRI, PTESFinder and CIRCexplorer achieved the best sensitivity. Consistent with the F1 measure, the same 5 methods still performed best in terms of AUC.

**Table 1:** Summary of accuracy measures on the positive and mixed datasets.

Datasets	Positive						Mixed					
Tools	#Detected	TP	S (%)	P (%)	F1	AUC	#Detected	TP	S (%)	P (%)	F1	AUC
CIRI	12,589	12,561	85.51	99.78	0.92	0.85	12,714	12,532	85.32	98.57	0.91	0.85
CF	9,460	9,383	63.88	99.19	0.78	0.64	9,513	9,383	63.88	98.63	0.78	0.63
DCC	8,558	8,512	57.95	99.46	0.73	0.58	8,562	8,512	57.95	99.42	0.73	0.58
FC	9,969	9,919	67.53	99.50	0.80	0.67	10,679	9,919	67.53	92.88	0.78	0.58
SG	13,470	12,766	86.91	94.77	0.91	0.84	14,158	12,340	84.01	87.16	0.86	0.80
CE	11,465	11,431	77.82	99.70	0.87	0.78	11,467	11,431	77.82	99.69	0.87	0.78
MS	9,072	9,032	61.49	99.56	0.76	0.61	9,075	9,028	61.46	99.48	0.76	0.61
UB	9,265	8,816	60.02	95.15	0.74	0.59	9,178	8,161	55.56	88.92	0.68	0.5
KNIFE	13,577	13,531	**92.12**	99.66	**0.96**	**0.92**	13,923	13,404	**91.25**	96.27	**0.94**	**0.87**
PF	12,341	12,303	83.76	99.69	0.91	0.84	12,357	12,298	83.72	99.52	0.91	0.83
NCLS	7,744	7,740	52.69	**99.95**	0.69	0.53	7,747	7,743	52.71	**99.95**	0.69	0.53

While the positive dataset contains only reads from circRNA species, the mixed dataset encompasses a large background dataset of simulated reads from RefSeq mRNAs, additionally. There are a total of 14,689 circRNA species in these 2 datasets. The total number of candidates and true positives predicted by each tool were calculated. AUC, Area under Precision-Recall Curve; CE, CIRCexplorer; CF, circRNA_finder; circRNA, circular RNA; F1, F1 score; FC, find_circ; MS, MapSplice; NCLS, NCLScan; P, precision; PF, PTESFinder; RefSeq, NCBI Reference Sequence database; RNase R, exonuclease that digests linear RNAs but preserves circRNAs; S, sensitivity; SG, Segemehl; TP, true positives; UB, UROBORUS.

### Evaluation with the background dataset

The background dataset contained only reads from linear RefSeq mRNAs; therefore, the number of candidates and supporting read counts reported, which could be directly accessed from each tool’s output, served as an indicator of false positive rate (Table 2). Here, NCLScan, MapSplice, CIRCexplorer, DCC, and PTESFinder tended to have a low false-positive rate, whereas Segemehl, find_circ and UROBORUS yielded the worst performance.

**Table 2:** Overview of circRNA candidates detected on the background dataset.

Datasets	Background	
Tools	#Detected	#total read counts
CIRI	150	633
CF	51	175
DCC	4	16
FC	712	14,220
SG	1,084	3,420
CE	2	24
MS	1	10
UB	620	3,476
KNIFE	554	3,960
PF	18	93
NCLS	0*	0*

The large background dataset contains only reads generated from RefSeq mRNAs, thus the false positive rate of a tool on this dataset correlates with the number of candidates it predicts. The total number of candidates and supporting-junction reads reported by each tool are provided here. CE, CIRCexplorer; CF, circRNA_finder; FC, find_circ; MS, MapSplice; NCLS, NCLScan; PF, PTESFinder; RefSeq, NCBI Reference Sequence database; SG, Segemehl; UB, UROBORUS.

*Notably, NCLscan aborted for failing to construct putative noncollinear references, therefore the number of candidates predicted by this tool was supposed to be 0 on this dataset.

### Evaluation with the mixed dataset

**Fig 2:**
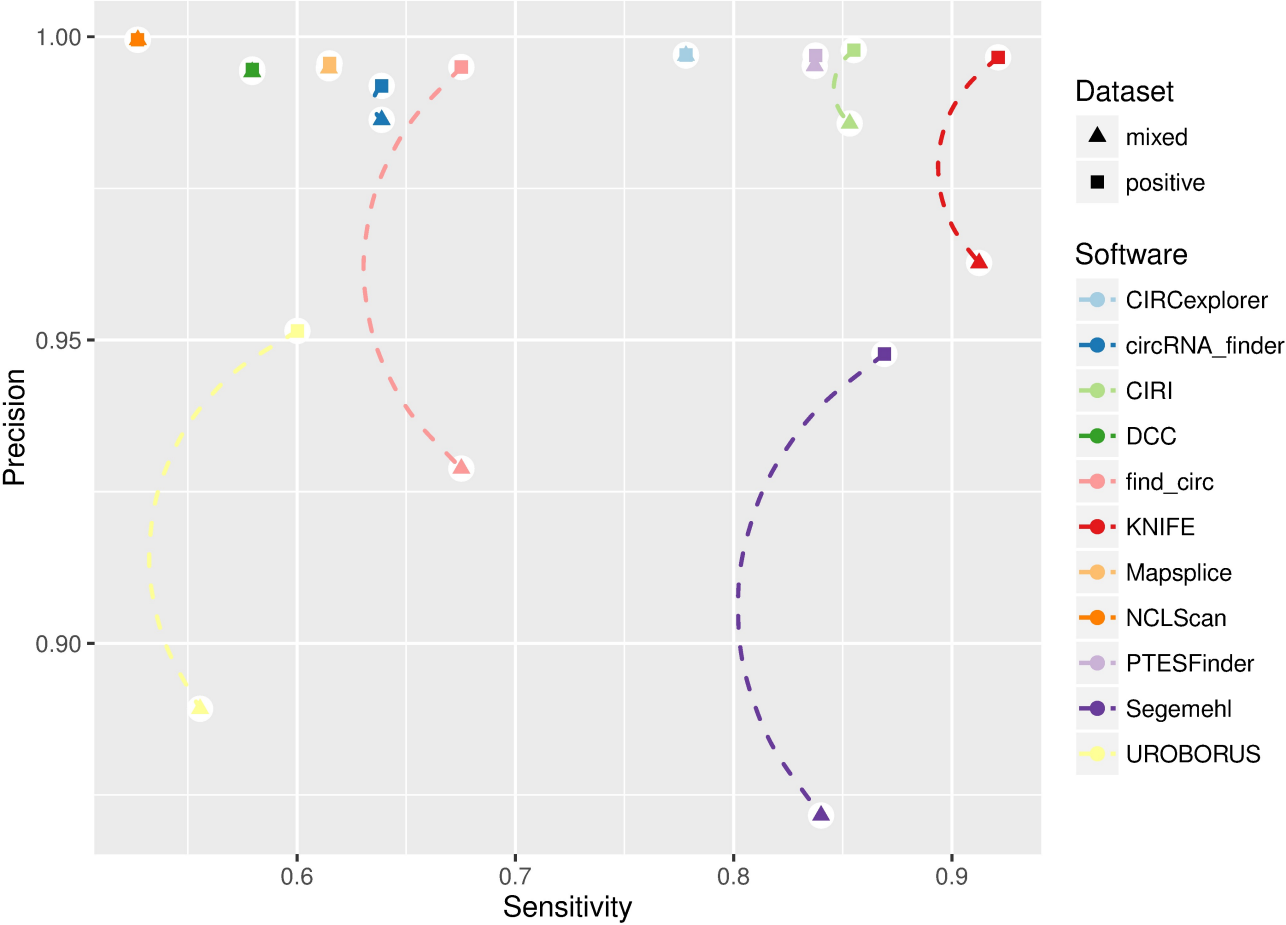
Comparison of performance on the synthetic positive and mixed datasets in terms of sensitivity and precision rate. After filtering for candidates with ≥2 supporting reads, the number of candidates and true positives detected by each method were computed, then precision and sensitivity rate for each method were depicted.

In the mixed dataset, we have 14,689 true positives. Similar to the positive dataset, metrics like precision, sensitivity, F1 measure, and AUC can be applied to evaluate the tools’ performance on this dataset. The results are presented in Table 1. We can see that NCLScan maintains the highest precision, while KNIFE, CIRI, PTESFinder, CIRCexplorer, and Segemehl exhibit the best with regard to F1 measure. Compared with the findings on the positive dataset, as shown in Fig 2, considerable drops of precision rate (−7.61%, −6.62%, and −6.23%) were observed for Segemehl, find_circ, and UROBORUS, respectively, indicating that their performances were vulnerable to background noise. Meanwhile, KNIFE, CIRI, and circRNA_finder also suffered minor loss of precision (−3.39%, −1.21%, and −0.56%, respectively). On the other hand, small decreases of sensitivity (−4.46%, −2.90%, and −0.87%) were only observed for UROBORUS, Segemehl, and KNIFE. Notably, NCLScan, CIRCexplorer, DCC, Mapsplice, and PTESFinder were robust to background noise, showing no pronounced reductions in precision or sensitivity rate. Fig 1B shows the influence on performance of each method when increasing the threshold for supporting read counts on this dataset. In general, NCLScan and CIRCexplorer dominated other tools regarding the precision measure, while KNIFE, CIRI, Segemehl, PTESFinder, and CIRCexplorer continued to be more sensitive than the rest of the tools. Of special note, except NCLScan, DCC, and MapSplice, the precision of all the other tools dropped to 0 in the end, because of the highly expressed false positives reported. The highest AUC achieved on this dataset was KNIFE (0.87), followed by CIRI (0.85), PTESFinder (0.83), Segemehl (0.80), and CIRCexplorer (0.78).

### Evaluation with the real datasets

For the real datasets, we found that the number of circRNA candidates detected correlated with the types of candidates they were able to detect. Generally, methods that were able to detect exonic, intronic, and intergenic circRNAs reported more candidates than tools that were limited to detect exonic and intronic circRNAs, but the tools that could detect exonic and intronic circRNAs predicted more candidates than tools that only reported exonic circRNAs, with the exception that KNIFE and PTESFinder tended to be sensitive at detecting exonic circRNAs (Table 3). Since we had no information about the true or false circRNA candidates detected in these samples, we mainly assessed each method’s performance from 4 perspectives: First, we calculated and compared the percentage of circRNA candidates detected by each method that were not depleted after RNase R treatment; though true circRNAs may not be enriched after RNase R treatment, we assumed that the higher the percentage a method achieves, the more reliable the method is. Second, for a specific sample, we calculated the proportions of circRNA candidates detected by a particular method that were also detected by every other method. Third, we assessed the sensitivity of each method at reads level, i.e., the number of backspliced junction reads per circRNA each method can recover. Fourth, we manually compiled a list of 282 circRNAs validated from 17 studies and checked how many of these verified circRNAs were detected by each method.

**Table 3:** Comparison of circRNA candidates detected with and without RNase R treatment.

Dataset	HeLa						Hs68					
Tools	RNaseR−	RNaseR+	Not depleted	Percentage (%)	Top 10 Enriched	Top 100 Not Depleted	RNaseR−	RNaseR+	Not depleted	Percentage (%)	Top 10 Enriched	Top 100 Not Depleted
CIRI	5,923	8,699	**3,210**	54.20	0	72	4,893	29,981	**3,400**	69.49	5	73
CF	3,448	5,190	1,597	46.32	1	72	3,577	24,060	2,094	58.54	5	72
DCC	3,892	4,781	1,760	45.22	1	71	3,340	19,746	2,107	63.08	5	75
FC	5,655	6,113	2,092	36.99	0	48	3,978	23,027	2,377	59.75	6	64
SG	17,504	13,973	2,506	14.32	0	45	35,253	77,702	3,094	8.78	0	7
CE	2,771	4,428	1,388	50.09	1	73	2,708	20,747	1,856	68.54	5	79
MS	3,256	4,780	1,765	**54.21**	0	66	2,429	18,197	1,854	**76.33**	**7**	**80**
KNIFE	4,643	5,295	2,055	44.26	0	**75**	3,546	19,491	2,359	66.53	6	76
PF	5,762	5,646	2,054	35.65	0	65	3,909	19,778	2,474	63.29	5	74
NCLS	2,117	2,832	954	45.06	1	65	1,378	11,001	892	64.73	**7**	78
UB	2,455	2,365	761	31.00	0	54	1,414	2,906	279	19.73	0	18

Having obtained candidates with ≥2 supporting reads, we normalized the supporting read counts with sequencing depth and defined a candidate as “not depleted” or “significantly enriched” if its normalized read counts were not reduced or had ≥5 folds of enrichment after RNase R treatment, respectively. The percentage of “not depleted” candidates in the RNase R–untreated sample is provided. Among the top 10 and top 100 most highly expressed candidates, the number of candidates “significantly enriched” or “not depleted” after RNase R treatment are also provided. In this table, these tools are ordered and divided into 3 groups, according to the types (genomic origin) of candidates that they are able to detect, namely: the first 5 are tools are able to detect exonic, intronic, and intergenic circRNA candidates, followed by CIRCexplorer and MapSplice, which are limited to predicting exonic and intronic candidates, with the last 4 methods predicting only exonic candidates. CE, CIRCexplorer; CF, circRNA_finder; circRNA, circular RNA; FC, find_circ; MS, MapSplice; NCLS, NCLScan; PF, PTESFinder; RNaseR-, sample without RNase R treatment; RNaseR+, sample with RNase R treatment; SG, Segemehl; UB, UROBORUS.

#### Percentage of circRNA candidates that were not depleted after RNase R treatment

After filtering for circRNA candidates with ≥2 supporting read counts, we normalized the backspliced junction read counts by sequencing depth [28, 39]. Similar to Hansen et al. [37], the ratio of normalized read counts between RNase R–treated and untreated samples was calculated. As shown in Table 3, with approximately equal sequencing depth, RNase R treatment indeed enabled the detection of many more candidates on the Hs68 samples. This is also confirmed on the HeLa samples. Although less than half the sequencing depth of the RNase R–untreated sample, a much larger number of candidates were detected by all the tools except PTESFinder, Segemehl, and UROBORUS on the HeLa RNase R–treated sample. In addition, we found that, on both HeLa and Hs68 samples, while MapSplice was capable of recovering the largest proportion of “not depleted” candidates, CIRI was much more sensitive to detecting such candidates and ranked second regarding the proportion of “not depleted” candidates. CIRCexplorer also exhibited decent performance and was ranked third in this analysis. When we focused on the top 100 most highly expressed candidates, as in Hansen et al. [37], we found that, while find_circ, UROBORUS, and Segemehl exhibited a relatively poor performance, most tools performed similarly, with a close percentage of “not depleted” candidates detected on HeLa (65%∼75%) and Hs68 (72%∼80%) samples.

#### Proportions of circRNA candidates detected by a specific method that were also detected by every other method

**Fig 3:**
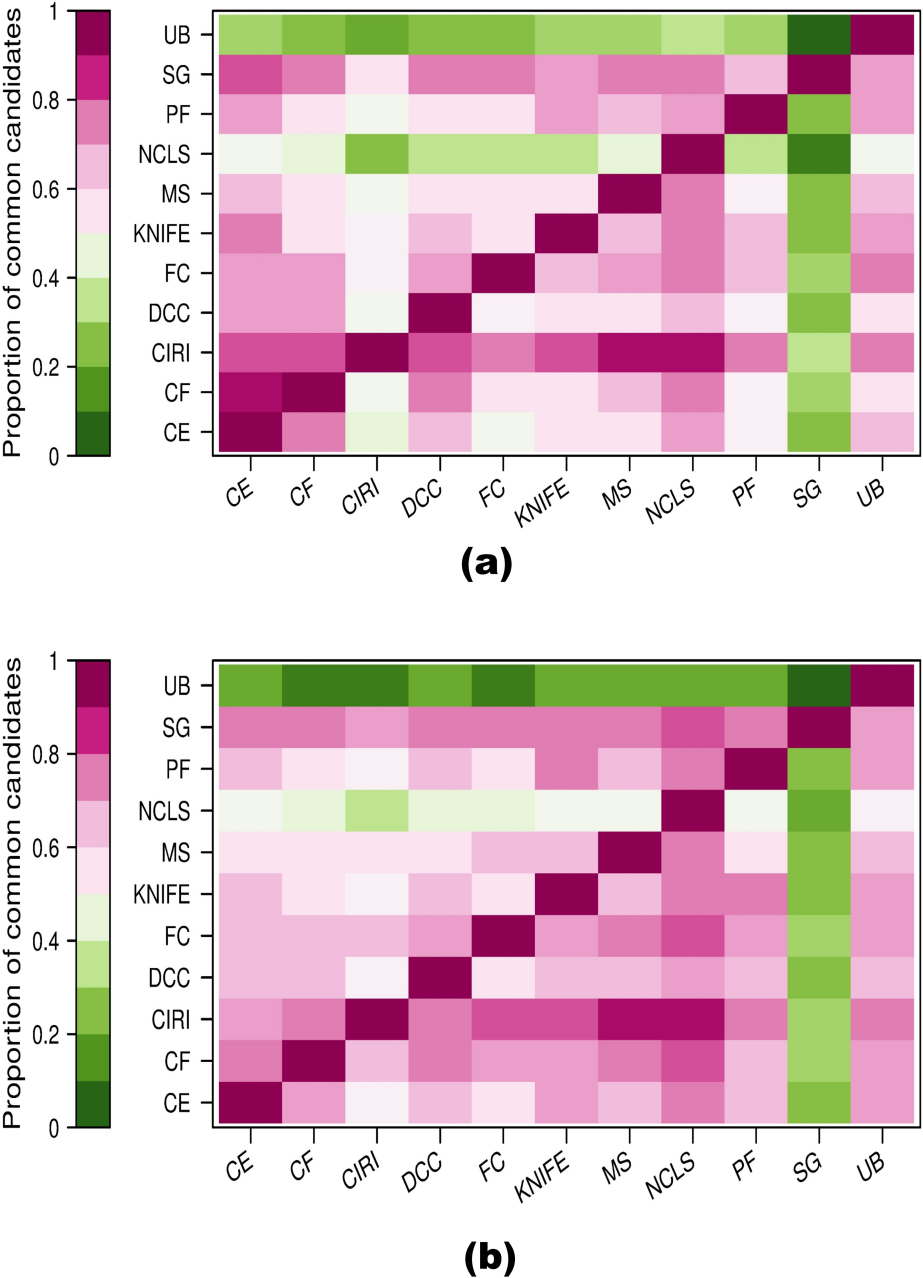
Coverage between circRNA detection methods on **(a)** HeLa and **(b)** Hs68 RNase R–treated data. For a pair of methods (i, j), the number of candidates detected by each method and the common candidates between them are calculated, then the proportion of common candidates for each method can be further computed and depicted. Cells within the same column reflect proportions of candidates detected by a specific method (column name) covered by other methods (row names) while cells within the same row show the proportions of candidates detected by other methods (column names) covered by a specific method (row name). CE, CIRCexplorer; CF, circRNA_finder; circRNA, circular RNA; FC, find_circ; MS, MapSplice; SG, Segemehl; NCLS, NCLScan; PF, PTESFinder; RNase R, exonuclease that digests linear RNAs but preserves circRNAs; UB, UROBORUS.

Mathematically, for all the methods M, ∀ *i*,*j* ∈ *M*, if we assume *N*_*i*_ and *N*_*j*_ as the total number of candidates detected by method *i* and *j*, respectively, and *C*(*i*,*j*) as the common candidates detected by both methods, then for method *i*, the proportion of common candidates is *P*(*i*,*j*) = *C*(*i*,*j*)/*N*_*i*_, while for method *j*, the proportion of common candidates is *P*(*j*,*i*) = *C*(*i*,*j*)/*N*_*j*_. If a large proportion of candidates detected by one method are often detected by the other methods (i.e., ∃ *i* ∈ *M*, ∀ *j* ∈ *M*\{*i*}, we have *P*(*i*,*j*) ≥ *P*_*threshold*_, *e*.*g*., *P*_*threshold*_ = 0.5), then the method would tend to have high precision. On the other hand, if candidates detected by one method frequently overlap with a large proportion of candidates detected by the other methods (i.e., ∃ *i* ∈ *M*, ∀ *j* ∈ *M*\{*i*}, we have *P*(*j*,*i*) ≥ *P*_*threshold*_, *e*.*g*., *P*_*threshold*_ = 0.5), then the method is sensitive and probably includes many true positives. After filtering for candidates with ≥2 backspliced junction reads on HeLa and Hs68 RNase R–treated sample data, we assessed the proportion of candidates detected by a specific method that were also detected by every other method (i.e., ∀ *i*, ∀ *j* ∈ *M*\{*i*}, we calculate *P*(*i*,*j*)), and the results were shown in Fig 3. We found that NCLScan was a conservative method in that a high proportion of candidates detected by this method were frequently detected by other methods, while CIRI and Segemehl were sensitive methods in that a large percentage of candidates detected by other methods were frequently detected by these 2 methods, but Segemehl tended to sacrifice much more precision, because a relatively large portion of candidates detected by this method were frequently missed by the other methods. In the case of UROBORUS, its behavior seemed to depend on the dataset. The number of candidates detected on the Hs68 RNase R–treated sample by this method was markedly smaller than by the other methods; as a consequence, a large proportion of candidates detected by the other methods were left out by this method.

#### Sensitivity at reads level

**Fig 4:**
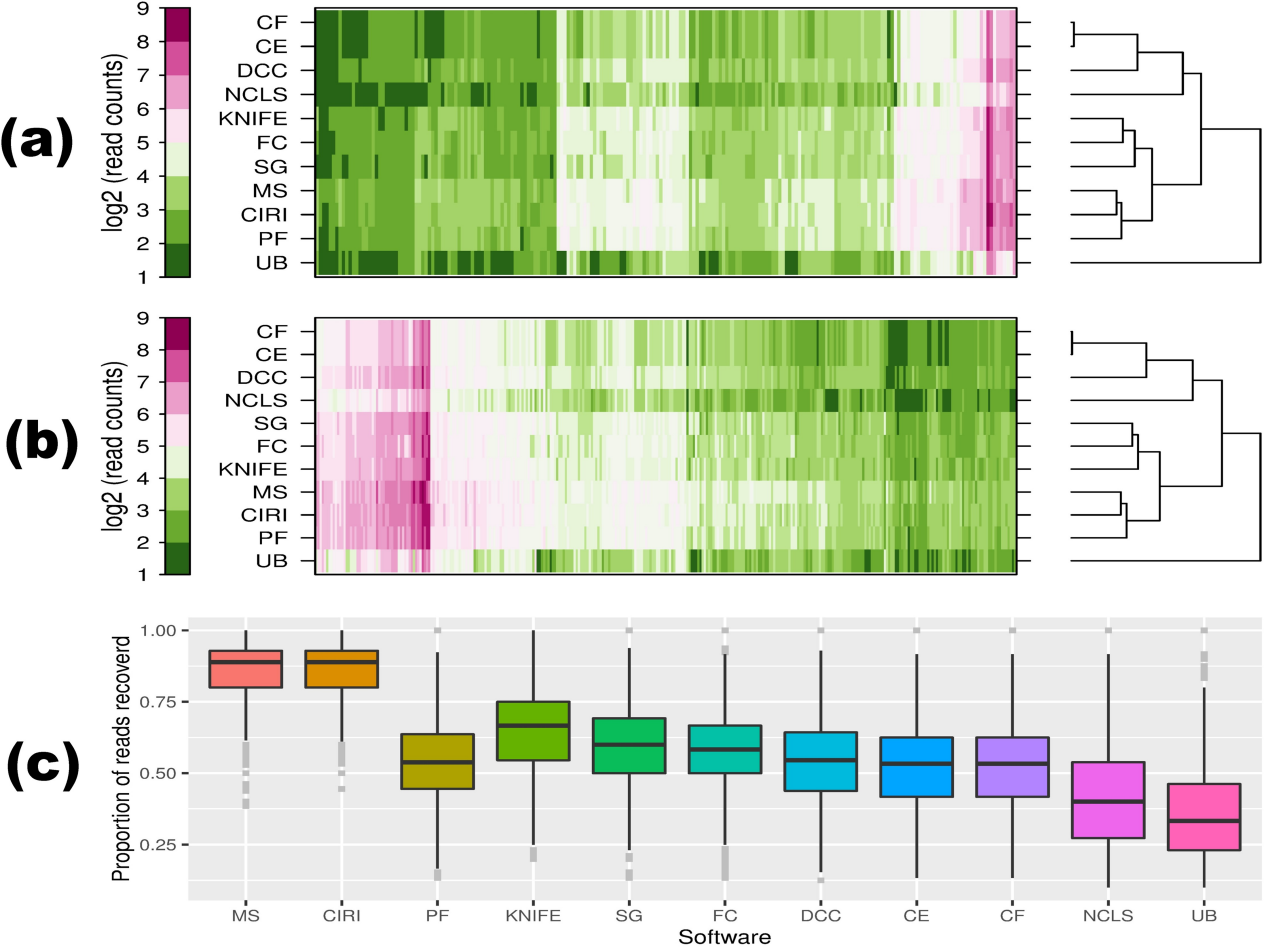
Sensitivity analysis at reads level. On **(a)** HeLa and **(b)** Hs68 RNase R–treated samples, common circRNA candidates detected by all the methods (659 and 903) and deposited in CircBase (608 and 724) were extracted, then candidates of which the spliced length is smaller than insert size of 500 base pairs (bp) and 400 bp, respectively, were further excluded (212 and 323). The numbers in parentheses above are candidates left after each filtering step. The number of supporting reads per candidate (log2 transformed) reported by each method was used in the cluster analysis. Each column represents a circRNA candidate and each row represents detection result of a specific method. The dendrogram was constructed via the average linkage hierarchical clustering approach, with intermediate Euclidean distance method chosen. **(c)** Backspliced junction reads recovery on positive dataset. After removing small-size candidates (smaller than insert size of 350 bp), proportion of backspliced junction reads recovered per candidate for the remnant common candidates was calculated for each method, and the results were used to depict the boxplot. circRNA, circular RNA; RNase R, exonuclease that digests linear RNAs but preserves circRNAs.

CircRNAs that are relatively abundant at a specific condition or developmental stage may possess important functions, and the number of backspliced junction reads is often used to quantify the expression level of circRNAs [3, 24, 27, 34–36, 38]. Therefore, the tools that are able to recover more of such reads will better serve this purpose. Of special note, for paired-end data, when both mates from a read pair span the same backspliced junction, which can be the case for small circRNAs, different tools undertake different counting methods. To our knowledge, CIRI, circRNA_finder, CIRCexplorer, KNIFE, DCC, and NCLScan take it once, while find_circ, MapSplice, PTESFinder, Segemehl, and UROBORUS count them twice. In our analysis, we focused on circRNA candidates detected from RNase R–treated samples. As shown in Fig 4A and 4B, consistent results were achieved on these 2 samples. Basically, these tools can be clustered into 4 groups according to their sensitivity. MapSplice, CIRI, and PTESFinder were in the most sensitive group, followed by the group of KNIFE, find_circ, and Segemehl. While CIRCexplorer, circRNA_finder, DCC (all of which are based on the STAR aligner), and NCLscan formed the third group, followed by the outlier of UROBORUS. In addition, the result was corroborated by similar analysis on the positive dataset (Fig 4C). Of note, probably due to the relatively high error rate (1%) introduced into the synthetic reads on the positive dataset and the strict filtering step applied by PTESFinder (no mismatches or indels within “n” nucleotides either side of the junction position, *n* = 10 in this case), the performance of PTESFinder on this dataset was not as sensitive as suggested from the real datasets. This indicates that its performance was vulnerable to sequencing error and depended on the sequencing depth.

#### Sensitivity for compiled experimentally validated circRNAs

**Fig 5:**
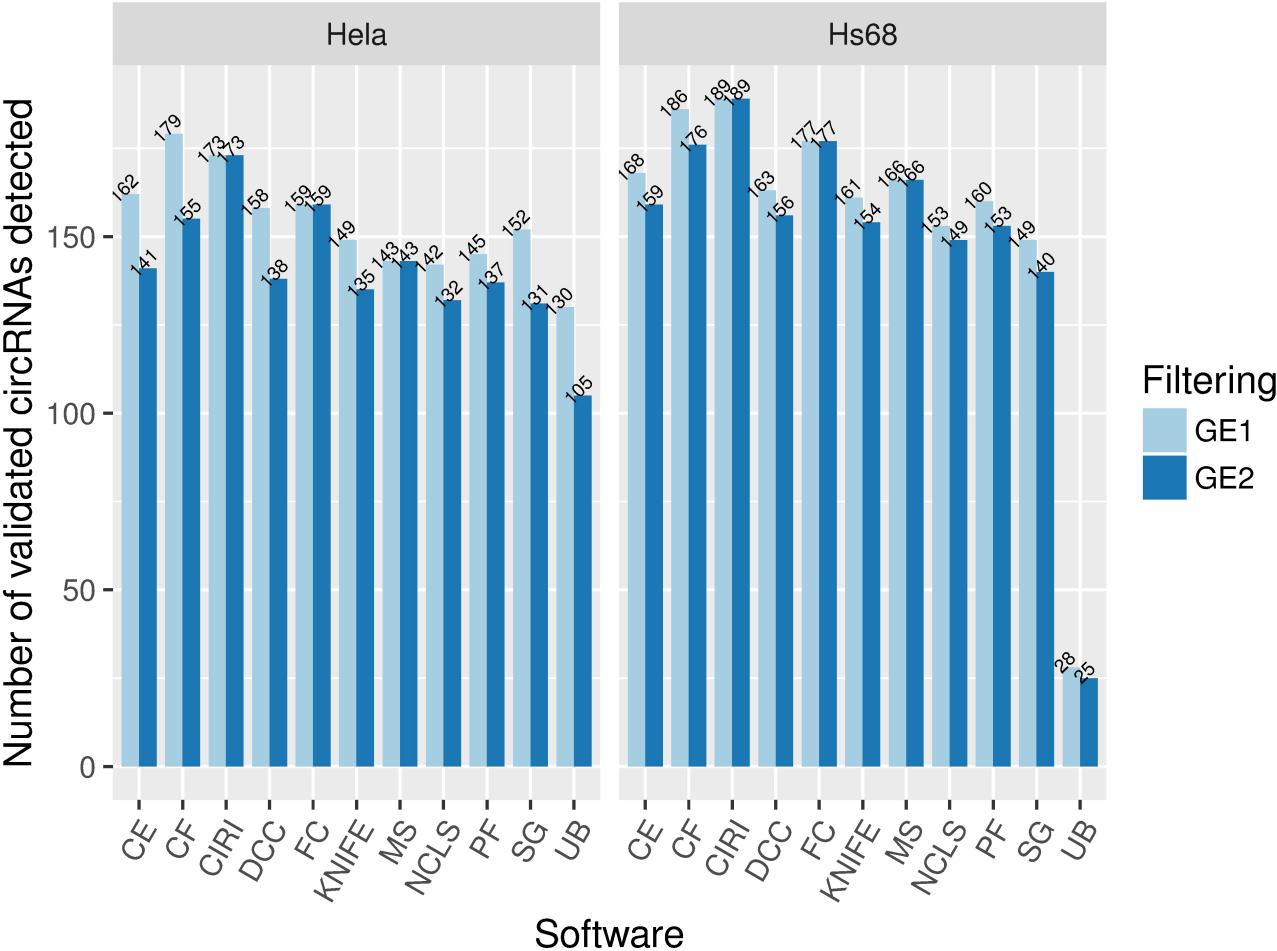
Number of experimentally verified circRNAs detected by each method. A total of 282 experimentally verified circRNAs were manually compiled from 17 studies, and the number of circRNAs rediscovered by each method on HeLa and Hs68 RNase R–treated samples was computed. CircRNAs with ≥1 and ≥2 supporting reads are shown in light blue and deep blue color, respectively. GE1: candidates with greater than or equal to 1 supporting reads; GE2: candidates with greater than or equal to 2 supporting reads. circRNA, circular RNA; RNase R, exonuclease that digests linear RNAs but preserves circRNAs.

We performed a broad survey of the published circRNA-related articles, from which we compiled a total of 282 experimentally verified circRNAs from 17 studies. Notably, they included circRNAs detected in various tissues or cell lines at different developmental stages, so many of them may not be expressed in HeLa or Hs68 samples. The detection of these validated circRNAs by each method on HeLa and Hs68 RNase R–treated samples is shown in Fig 5. It shows that varying numbers of validated circRNAs were lost upon filtering for candidates with ≥2 supporting reads. In addition, CIRI was found to be the most sensitive method when we only considered circRNA candidates with ≥2 supporting backspliced junction reads.

### Computational cost overview

**Fig 6:**
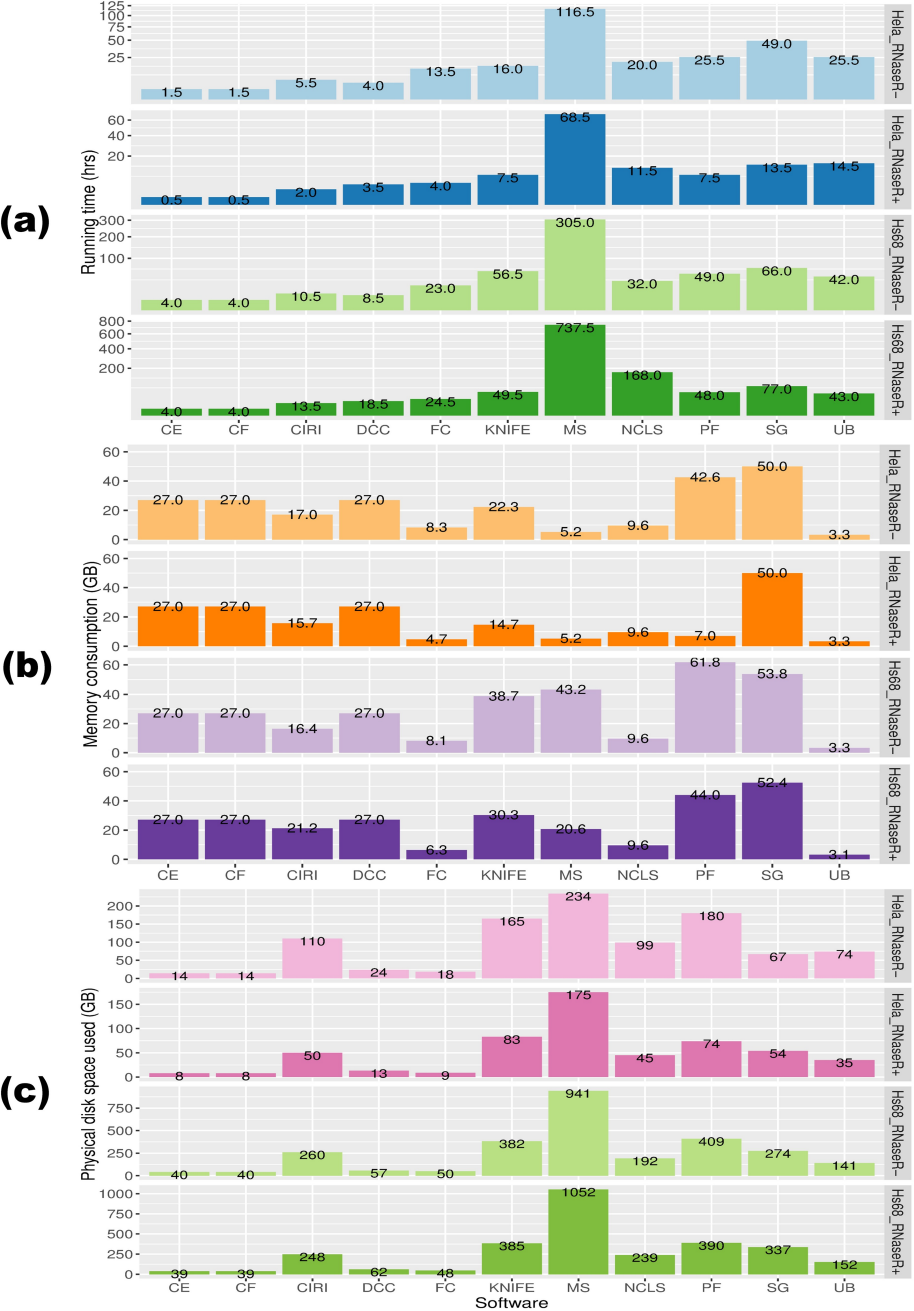
**(a)** Computational cost for each method on metrics of runtime, **(b)** memory consumption, and **(c)** physical disk space usage. While HeLa_RNaseR+ and HeLa_RNaseR− datasets are moderately sized RNA-Seq datasets, Hs68_RNaseR+ and Hs68_RNaseR− are examples of datasets with deep sequencing depth. Note: The analyses were run on an Ubuntu 10.04 server with two Intel® Xeon® E5530 Central Processing Units and 102 gigabytes of RAM. The running time presented was based on at most 3 threads allocated for each tool.

We evaluated the computational efficiency of each software package using the metrics of runtime, RAM consumption, and physical disk space. We found that the computational cost not only correlated with the sequencing depth, but was also affected by the abundance of circRNA candidates detected in each sample. As Fig 6A shows, when running on large datasets (i.e., Hs68_RNaseR− and Hs68_RNaseR+) with an equal number of 3 threads allocated, only CIRCexplorer, circRNA_finder, DCC, CIRI, and find_circ could finish within a day or so, while MapSplice took an incredibly long time of about 13 days and a month to finish Hs68_RNaseR− and Hs68_RNaseR+, respectively. The relative long runtime of MapSplice was also confirmed by the study of Hansen et al. [37]. For Central Processing Unit (CPU)-intensive tools such as MapSplice, we recommend users to run these tools on servers with adequate processors allocated to reduce running time. Regarding memory consumption, only UROBORUS and find_circ were capable of processing large datasets on a standard PC equipped with 8 gigabytes (GB) of RAM. NCLScan consistently required approximately 10 GB, while CIRCexplorer, circRNA_finder, and DCC needed about 27 GB to run the underlying STAR aligner. Also, Segemehl was the least efficient, demanding about 50 GB to run all the time. For other software packages, moderate or sharp increase of memory consumption was observed when the dataset shifted from moderate to large size (Fig 6B). In addition, MapSplice, PTESFinder, KNIFE, Segemehl, CIRI, and NCLScan were found to be the 6 least efficient software packages regarding physical disk space usage (Fig 6C), indicating that users should prepare adequate computational resources before running these pipelines on large datasets.

## Discussion

The global and accurate identification of circRNAs from RNA-Seq data serves as a fundamental step towards revealing their biogenesis and functions. Here, we provided an improved and easy-to-use circRNA read simulator that we believe will benefit the circRNA research community. Besides, we performed a comprehensive evaluation of 11 circRNA detection tools using synthetic and real datasets based on multiple metrics such as sensitivity, precision, F1 measure, AUC, RAM consumption, running time, and physical disk space used.

Taken together, we observed concordant results on the synthetic and real datasets. Generally, CIRI, CIRCexplorer, and KNIFE, which achieved better balanced performance between their precision and sensitivity, compared favorably to the other methods, whereas NCLScan and MapSplice were conservative methods with comparable precision but less favorable sensitivity. Conversely, Segemehl was sensitive but suffered with the presence of many false positives in the output. Together with find_circ and UROBORUS, these 3 methods exhibited the worst precision based on our comparisons with the background and real datasets. The performance of PTESFinder was noticeable on the synthetic dataset but less pronounced on the real datasets. Its performance tended to be variable depending on the dataset. Also, we found that CIRI and MapSplice were the most sensitive methods to recover backspliced junction reads for candidates detected. For the positive dataset used, it should be noted that we might have introduced bias into this dataset when we generated backspliced junction reads for candidates deposited in CircBase. Specifically, the HeLa circRNAs used were reported in the Salzman 2013 study [36], thus potential favor may be with KNIFE. But the high recall rate achieved by KNIFE on this dataset could also be attributed to its high sensitivity, as demonstrated in Szabo et al. [33]. For KNIFE, it also should be pointed out that the output from its de novo module was not incorporated in our study, thus the sensitivity of this tool may be underestimated in our analysis on real datasets.

In addition to the performance factor, practical issues may also affect the choice of an optimal tool. For instance, Hansen et al. failed to run KNIFE and Segemehl in their study [37]. In our experience, generally, the installation process would be more complicated for tools with more dependencies (Table 4). Also, great differences in computational cost were observed among these tools (Fig 6); practitioners need to pay attention to this when analyzing large datasets. Finally, a comprehensive manual and user-friendly output would be beneficial to the users. Though most tools included detailed user guides, some only provided limited descriptions (e.g., circRNA_finder and PTESFinder). Besides, the backspliced junction reads provided critical information for further scrutinizing the authenticity of a candidate of interest; however, only some of the tools (e.g., CIRI and KNIFE) included the identifiers of such reads in the output.

**Table 4:** Overview of circRNA detection tools evaluated.

Method	Approach		Genomic origin	Version	Reference	Dependencies
CIRI	Segmented read-based	Exonic, intronic, intergenic		2.0.1	[39]	Bwa, perl
circRNA_finder	Segmented read-based	Exonic, intronic, intergenic		NA	[34]	STAR, samtools, perl
DCC	Segmented read-based	Exonic, intronic, intergenic		0.3.2	[42]	STAR, python (pysam, pybedtools, numpy, pandas, HTSeq)
find_circ	Segmented read-based	Exonic, intronic, intergenic		1.0	[2]	Bowtie2, samtools, python (pysam, numpy)
Segemehl	Segmented read-based	Exonic, intronic, intergenic		0.2.0	[47]	samtools
CIRCexplorer	Segmented read-based	Exonic, intronic		1.1.5	[15]	STAR, bedtools, python (pysam, docopt, Interval)
MapSplice	Segmented read-based	Exonic, intronic		2.2.0	[48]	Bowtie, samtools, python
UROBORUS	Segmented read-based	Exonic		0.0.2	[38]	Bowtie, Bowtie2, tophat2, samtools, perl
KNIFE	Candidate-based	Exonic*		1.4	[33]	Bowtie, Bowtie2, samtools, python (numpy, scipy), R (data.table), perl
PTESFinder	Candidate-based	Exonic		1.0	[41]	Bowtie, Bowtie2, bedtools, Java
NCLScan	Candidate-based	Exonic		1.5	[40]	Bwa, Blat, Novoalign, bedtools, samtools, python

Note

*The de novo module in KNIFE can also detect circRNAs from unannotated spliced sites; these unannotated spliced sites may be located in the intronic or intergenic regions. circRNA, circular RNA; NA, not available, the version number of circRNA_finder tool was not provided by its developers.

Of all the methods evaluated here, no single tool dominates on all the metrics used, and there is still much space for further improvement regarding methods for the global detection of these noncollinear molecules from RNA-Seq data. For example, all of them were originally designed to identify circRNAs originating from the same gene locus, but a recent report [45] pointed out that circRNAs could derive from gene fusion events and potentially play a critical role in cancer pathogenesis. These fusion circRNAs are overlooked by the current methods, underscoring the complexity of the RNA world and the need to refine the existing methods or develop new ones for circRNA detection.

## Materials and methods

### Software packages for detecting circRNAs

To our knowledge, about 11 tools are now available for the detection of circRNAs from RNA-Seq data and can be broadly divided into two groups according to the underlying strategies to detect circRNAs [7, 10, 19] (Table 4). For instance, KNIFE, NCLScan, and PTESFinder all require that the putative circRNA sequences to be constructed with gene annotation information are provided in order to detect circRNAs. This strategy was called “pseudo-reference-based” in [7, 10] or “candidate-based” approach in [19]. However, the difference is that KNIFE directly constructs all the potential out-of-order exon–exon junction sequences from gene annotation information before alignment, while NCLScan and PTESFinder create the putative circRNA sequences levering the mapping information of the segmented anchors obtained after alignment to the genome or transcriptome. The other strategy that other tools used was called “fragmented-based” in [7, 10] or “segmented read approach” in [19], which identified backsplicing junctions from the mapping information of a multiple-split read’s alignment to the genome. Under this category, specifically circRNA_finder, CIRCexplorer, DCC, MapSplice, and Segemehl can be assigned to a subgroup, because they devise spliced alignment algorithms to detect and parse the backsplicing events, whereas find_circ and UROBORUS can be grouped together, as they both gather the unmapped reads after mapping them to the genome, extract the first and last 20 bp anchors from the unmapped reads, and then derive the backsplicing events from the mapping information of these anchors. Finally, CIRI is unique. It detects the paired chiastic clipping (PCC) signals from the mapping information of reads by local alignment with BWA-MEM [46] and combines with systematic filtering steps to remove potential false positives. For evaluation of their performance, these tools and associated software packages were deployed on an Ubuntu 10.04 server, equipped with 2 Intel(R) Xeon(R) E5530 CPUs and 102 GB of RAM. We followed the instructions and recommendations provided in their manuals and focused on output circRNAs with ≥2 backspliced junction reads. Here, we provide a brief summary of these software packages. For details of the algorithms underlying each tool, users can refer to the papers introducing each method.

circRNA_finder [34] requires paired-end sequencing data and relies on the RNA-Seq spliced alignment software STAR [49]. After read alignment, the output putative chimeric junction reads are filtered and collapsed into a set of putative circularization junctions based on the following restrictions: (1) At most, 3 mismatches are allowed, and only unique mapped reads are used. (2) The distance between the splice donor and acceptor should be less than 100 kilobases (kbs). (3) One read in a pair should span the backsplicing junction site, while the other should be mapped within the interval between the splice donor and acceptor. In this study, neither circRNA candidates without GT/AG splice sites nor those derived from mitochondria were taken into consideration.

CIRCexplorer [15] is a Python-based tool, providing user-friendly circRNA detection output. Initially, it uses TopHat [50] to do the spliced alignment of reads to genome, then extracts the unmapped reads to detect backsplicing events by alignment with TopHat-Fusion [51]. Reads that are split and mapped to the same chromosome but in reverse order are candidate backspliced junction reads. The mapping positions of these reads are realigned and adjusted if needed, in order that the donor and acceptor splice sites derived are consistent with canonical splice sites from known gene annotation. Currently, it also supports parsing the STAR spliced alignment output. Since TopHat and TopHat-Fusion require much more time to complete the alignment step, in this study, we took the intermediate STAR alignment output generated during running circRNA_finder as the input for CIRCexplorer. As a consequence, the computational costs (i.e., RAM, running time, and physical disk space) of these 2 pipelines were almost equivalent because the computational resources required by them were negligible during the circRNA detection phase compared with that in the process of read alignment.

Another software package that utilized STAR alignment software and is evaluated in this study is DCC [42]. However, to improve the detection of small circRNAs, in addition to the usual alignment of read pairs from paired-end data as a whole, the DCC pipeline also recommends that users run an alignment of each segment from read pairs separately. This almost doubles the computational cost during the alignment phase. During the process of detection, several filtering steps are applied: (1) If paired-end data are being dealt with, mapping of mates should match with the relevant circRNA. (2) If biological replicates are available, filtering for common circRNAs detected by these replicates would be allowed. (3) The canonical GT/AG splicing signal should be presented in the backsplicing junction borders. (4) Backsplicing events from mitochondria are discarded. (5) Candidate circRNAs from repetitive or homologous regions are removed.

Find_circ [2] is one of the 5 tools evaluated in this study that utilizes Bowtie2 [52] and/or Bowtie [52] to perform read alignment. In short, first, it collects the unmapped reads generated during the first round of alignment to the genome. Second, it extracts the first and last 20-bp anchors from each unmapped read to perform the second alignment. If the 2 anchors are mapped to positions within spliced exons in an opposite orientation, it indicates circRNA splicing. Third, it extends the anchors’ alignment, collects and outputs the identified splice junctions, and keeps those junction-spanning reads. Finally, it applies a series of filtering steps to check and report reliable circRNA candidates.

UROBORUS [38] is also a circRNA-detection pipeline based on the Bowtie RNA-Seq alignment tool. First, it employs TopHat to perform splice alignment. Second, it collects the first and last 20 bp of an unmapped read as anchors and realigns these anchors using TopHat to gather balanced mapped junctions and unbalanced mapped junctions. Third, these 2 types of junction-spanning anchors are separately handled to infer the potential backspliced junction reads. In the end, the reads obtained above are further aligned to the genome using Bowtie; those that map to the same chromosome but in reverse orientation are annotated as candidate circRNA-derived reads.

PTESFinder [41] employs both Bowtie and Bowtie2 to perform read alignment. It only detects backsplicing junctions stemming from known splice sites. Intriguingly, it does not make use of the paired-end information, even if it is available. The detection process can be summarized as follows. First, it extracts the first and last 20-bp anchors from a read and aligns them to transcriptome reference sequences. Second, it exploits the anchors’ mapping information to detect the exon-shuffling events and also generates the putative circRNA sequences flanking the backsplicing junction sites. Third, it aligns the original reads to the putative circRNA sequences, genome, and transcriptome. Finally, to eliminate potential false positives, it requires greater mapping scores obtained from the putative circRNA sequences than those from genome or transcriptome, and also user-adjustable criteria on mapped reads must be satisfied to support putative circRNA sequences.

KNIFE [33] starts by mapping reads to the genome, rRNA sequences, transcriptome, and customized linear and backspliced junction databases separately, with the help of Bowtie2. It discards possible backspliced junction reads when they also map with high scores to the other databases mentioned above. For those remaining backspliced junction-spanning reads, it further categorizes them into circRNA and decoy reads based on the mapping information of the mate when paired-end data are available. Finally, for reads aligned to none of the databases mentioned above, it remedies with a de novo analysis module to detect circRNAs derived from unannotated splice sites. However, the break points of these inferred circRNAs are window-/bin-based; in other words, they are not exact break points, so we did not incorporate these circRNA candidates into this study. The major advantage of KNIFE over other tools, according to its developers, is the method of filtering circRNAs for which there is high confidence. It employs a statistical framework to obtain the posterior probability of every circular read collected to subsequently predict whether it is true or false positive.

Unlike the above circRNA detection tools based on Bowtie, which require extracting a fixed size of anchors from the unmapped reads to identify potential backspliced junctions, the underlying BWA-MEM aligner of CIRI [39] can automatically determine the break points of query reads derived from circRNAs. After BWA-MEM alignment, CIRI scans the alignment results twice. Briefly, during the first scan, it collects the PCC signals supporting the backsplicing junctions and appropriate paired-end mapping signals consistent with the circRNA templates. Then, it checks and filters for those junction signals with canonical GT/AG splice sites (if a gene annotation file is provided, other possible splice-site signals flanking exon boundaries in this file will also be considered). During the second scan, it further clusters the unbalanced junction reads missed during the first scan by applying a dynamic programming alignment algorithm and also filters out those potential false positive junctions derived from repetitive or homologous regions.

MapSplice [48] is one of the 3 software packages evaluated in this study that are able to identify multiple types of splice junction events. Specifically, it’s de novo splice mapping software that can segment reads into multiple anchors to detect canonical and noncanonical junctions in RNA-Seq data. This algorithm was applied in the study by Jeck et al. [3] and detected more than 25,000 distinct circRNA species in human fibroblasts that were resistant to RNase R. This tool is memory efficient when running on an RNA-Seq dataset with a regular sequencing depth but takes longer to run than all of the other packages presented here when equivalent numbers of threads are allocated.

Segemehl [47] is also a multi-split RNA-Seq mapping tool that can identify circRNA, canonical splicing, trans-splicing, and gene fusion events. It is claimed to be more sensitive than its counterparts at detecting these events. Notably, Segemehl consumes a large amount of RAM when the reference genome is large, for example, approximately 50 GB for the human genome. In such cases, runs on computers with a small memory allocation will fail.

NCLscan [40] is another RNA-Seq analysis tool that is claimed to be accurate at identifying noncollinear transcripts such as trans-splicing, fusion, and circRNAs from transcriptome data. One of the key steps in this pipeline is to construct the putative noncollinear references with gene annotation information and BLAT alignment output of the concatenated sequences from unmapped read pairs. To eliminate false positives, it undertakes several stepwise alignments and filtering, integrating different aligners such as BWA [53], BLAT [54], and license-required Novoalign (www.novocraft.com).

### Datasets used

Table 5 provides a summary of all of the datasets used. Detailed descriptions of these datasets are provided below.

**Table 5:** Summary of the datasets used in this study.

Datasets	Library construction	Sequencing type	#Read pairs	SRA accession number
Positive	/	Simulated PE101	1,071,113	/
Background	/	Simulated PE101	168,552,417	/
Mixed	/	Simulated PE101	169,623,530	/
HeLa_RNaseR−	rRNA(−)	Illumina PE101	80,533,660	SRR1637089 and SRR1637090
HeLa_RNaseR+	rRNA(−) and RNaseR(+)	Illumina PE101	36,758,130	SRR1636985 and SRR1636986
Hs68_RNaseR−	rRNA (−)	Illumina PE100	202,521,855	SRR444975
Hs68_RNaseR+	rRNA(−) and RNaseR(+)	Illumina PE100	196,383,303	SRR445016

HeLa_RNaseR−, HeLa cells without RNase R treatment; HeLa_RNaseR+, HeLa cells with RNase R treatment; Hs68_RNaseR−, Hs68 cells without RNase R treatment; Hs68_RNaseR+, Hs68 cells with RNase R treatment; PE101, Paired-End 101 bp sequencing; PE100, Paired-End 100 bp sequencing; SRA, NCBI Sequence Read Archive database.

#### Positive dataset

The positive dataset contains a total of 1,071,113 pairs of synthetic reads, with the sequence length of 101 bp and insert size of 350 bp. These synthetic data encompass a total of 14,689 circRNA species. The number of backspliced junction read pairs supporting each circRNA ranges from 2 to 24, while the size of circRNAs varies from 51 to 846,530 bp. This simulated positive dataset was generated by an improved circRNA read simulation tool named CIRI-simulator [39], which was originally released with CIRI. To accurately generate mimicking circRNA reads, we overhauled this circRNA simulation tool. It now supports generating circRNAs deposited in CircBase, which are far more appropriate circRNA candidates than those generated from the joining of 2 randomly chosen out-of-order exons. A total of 14,689 circRNA species were produced from those reportedly detected in HeLa cells. This accurate and easy-to-use simulation tool, which we believe will benefit the circRNA research community, can be accessed at: https://github.com/linatbeishan/circRNA_detection_review.

#### Background dataset

Simulated paired-end RNA-Seq data were generated with a widely used read simulator ART [55]. Briefly, the RefSeq mRNA sequences were downloaded from the UCSC Genome Browser first, then the simulator was executed using the downloaded sequences as input database. Indel and substitution variants were introduced into the generated reads. Specifically, to take the influence of poor sequencing quality into consideration, we shifted down the quality score of reads to increase substitution sequencing errors. Finally, a large negative dataset with sequencing length of 101 bp and insert size of 350 ± 10 bp was generated (the command used: art_illumina -ss HS25 -d simulate -na -i refMrna.fa -o simulate -l 101 -f 200 -p -m 350 -s 10 -sp -rs 20160830 -qs -13 -qs2–13).

#### Mixed dataset

The mixed dataset was generated by combining the background and positive datasets to further evaluate the performance of each method.

#### Real datasets

We included 6 runs of real datasets produced in 2 separate independent circRNA-related studies [3, 39]. The first is the HeLa cell-line dataset, which was also used in Chen et al. [7]. This dataset comprises 4 runs of rRNA-depleted RNA-Seq libraries downloaded from the NCBI Sequence Reads Archive (accession numbers SRR1636985, SRR1636986, SRR1637089, and SRR1637090). Specifically, SRR1636985 and SRR1636986 are from samples further treated with RNase R enzyme after rRNAs had been depleted. Therefore, we combined SRR1636985 and SRR1636986 as a HeLa_RNaseR+ sample and SRR1637089 and SRR1637090 as a HeLa_RNaseR− sample. After cleaning the raw data, there were approximately 80.5 million and 36.8 million PE101 read pairs left for HeLa_RNaseR− and HeLa_RNaseR+, respectively. Furthermore, to eliminate possible bias from data generated by a single group and assess the performance of each method on large datasets, we incorporated another 2 runs of deep sequencing PE100 RNA-Seq data derived from Hs68 cells (accession numbers SRR444975 and SRR445016), which were also used in [37]. Both runs were similar in being rRNA-depleted, but SRR445016 was from samples additionally treated with RNase R enzyme. After cleaning, the numbers of remaining read pairs for SRR444975 and SRR445016 were approximately 202.5 million and 196.4 million, respectively.

## References

[1] Nigro JM, Cho KR, Fearon ER, Kern SE, Ruppert JM, Oliner JD, et al Scrambled exons. Cell. 1991;64(3):607–13. 1991322 10.1016/0092-8674(91)90244-s

[2] Memczak S, Jens M, Elefsinioti A, Torti F, Krueger J, Rybak A, et al Circular RNAs are a large class of animal RNAs with regulatory potency. Nature. 2013;495(7441):333–8. doi: 10.1038/nature11928 23446348 10.1038/nature11928

[3] Jeck WR, Sorrentino JA, Wang K, Slevin MK, Burd CE, Liu J, et al Circular RNAs are abundant, conserved, and associated with ALU repeats. Rna. 2013;19(2):141–57. PubMed Central PMCID: PMC3543092. doi: 10.1261/rna.035667.112 23249747 10.1261/rna.035667.112 PMC3543092

[4] Salzman J, Gawad C, Wang PL, Lacayo N, Brown PO. Circular RNAs are the predominant transcript isoform from hundreds of human genes in diverse cell types. PloS ONE. 2012;7(2):e30733 PubMed Central PMCID: PMC3270023. doi: 10.1371/journal.pone.0030733 22319583 10.1371/journal.pone.0030733 PMC3270023

[5] Lu T, Cui L, Zhou Y, Zhu C, Fan D, Gong H, et al Transcriptome-wide investigation of circular RNAs in rice. Rna. 2015;21(12):2076–87. PubMed Central PMCID: PMC4647462. doi: 10.1261/rna.052282.115 26464523 10.1261/rna.052282.115 PMC4647462

[6] Chen L-L. The biogenesis and emerging roles of circular RNAs. Nature Reviews Molecular Cell Biology. 2016;17(4):205–11. doi: 10.1038/nrm.2015.32 26908011 10.1038/nrm.2015.32

[7] Chen I, Chen CY, Chuang TJ. Biogenesis, identification, and function of exonic circular RNAs. Wiley interdisciplinary reviews RNA. 2015;6(5):563–79. doi: 10.1002/wrna.1294 26230526 10.1002/wrna.1294 PMC5042038

[8] Hansen TB, Kjems J, Damgaard CK. Circular RNA and miR-7 in cancer. Cancer research. 2013;73(18):5609–12. doi: 10.1158/0008-5472.CAN-13-1568 24014594 10.1158/0008-5472.CAN-13-1568

[9] Zhao ZJ, Shen J. Circular RNA participates in the carcinogenesis and the malignant behavior of cancer. RNA biology. 2015:1–8. 10.1080/15476286.2015.1122162 PMC5449088 26649774

[10] Meng X, Li X, Zhang P, Wang J, Zhou Y, Chen M. Circular RNA: an emerging key player in RNA world. Briefings in bioinformatics. 2016. 10.1093/bib/bbw045 27255916

[11] Lasda E, Parker R. Circular RNAs: diversity of form and function. Rna. 2014;20(12):1829–42. PubMed Central PMCID: PMC4238349. doi: 10.1261/rna.047126.114 25404635 10.1261/rna.047126.114 PMC4238349

[12] Hansen TB, Wiklund ED, Bramsen JB, Villadsen SB, Statham AL, Clark SJ, et al miRNA-dependent gene silencing involving Ago2-mediated cleavage of a circular antisense RNA. The EMBO journal. 2011;30(21):4414–22. PubMed Central PMCID: PMC3230379. doi: 10.1038/emboj.2011.359 21964070 10.1038/emboj.2011.359 PMC3230379

[13] Hansen TB, Jensen TI, Clausen BH, Bramsen JB, Finsen B, Damgaard CK, et al Natural RNA circles function as efficient microRNA sponges. Nature. 2013;495(7441):384–8. doi: 10.1038/nature11993 23446346 10.1038/nature11993

[14] Ashwal-Fluss R, Meyer M, Pamudurti NR, Ivanov A, Bartok O, Hanan M, et al circRNA biogenesis competes with pre-mRNA splicing. Molecular cell. 2014;56(1):55–66. doi: 10.1016/j.molcel.2014.08.019 25242144 10.1016/j.molcel.2014.08.019

[15] Zhang XO, Wang HB, Zhang Y, Lu X, Chen LL, Yang L. Complementary sequence-mediated exon circularization. Cell. 2014;159(1):134–47. doi: 10.1016/j.cell.2014.09.001 25242744 10.1016/j.cell.2014.09.001

[16] Zhang Y, Zhang XO, Chen T, Xiang JF, Yin QF, Xing YH, et al Circular intronic long noncoding RNAs. Molecular cell. 2013;51(6):792–806. doi: 10.1016/j.molcel.2013.08.017 24035497 10.1016/j.molcel.2013.08.017

[17] Li Z, Huang C, Bao C, Chen L, Lin M, Wang X, et al Exon-intron circular RNAs regulate transcription in the nucleus. Nature structural & molecular biology. 2015;22(3):256–64. 10.1038/nsmb.2959 25664725

[18] Capel B, Swain A, Nicolis S, Hacker A, Walter M, Koopman P, et al Circular transcripts of the testis-determining gene Sry in adult mouse testis. Cell. 1993;73(5):1019–30. 7684656 10.1016/0092-8674(93)90279-y

[19] Jeck WR, Sharpless NE. Detecting and characterizing circular RNAs. Nature biotechnology. 2014;32(5):453–61. PubMed Central PMCID: PMC4121655. doi: 10.1038/nbt.2890 24811520 10.1038/nbt.2890 PMC4121655

[20] Zaphiropoulos PG. Exon skipping and circular RNA formation in transcripts of the human cytochrome P-450 2C18 gene in epidermis and of the rat androgen binding protein gene in testis. Molecular and cellular biology. 1997;17(6):2985–93. PubMed Central PMCID: PMC232150. 9154796 10.1128/mcb.17.6.2985 PMC232150

[21] Suzuki H, Zuo Y, Wang J, Zhang MQ, Malhotra A, Mayeda A. Characterization of RNase R-digested cellular RNA source that consists of lariat and circular RNAs from pre-mRNA splicing. Nucleic acids research. 2006;34(8):e63 PubMed Central PMCID: PMC1458517. doi: 10.1093/nar/gkl151 16682442 10.1093/nar/gkl151 PMC1458517

[22] Enuka Y, Lauriola M, Feldman ME, Sas-Chen A, Ulitsky I, Yarden Y. Circular RNAs are long-lived and display only minimal early alterations in response to a growth factor. Nucleic acids research. 2016;44(3):1370–83. PubMed Central PMCID: PMC4756822. doi: 10.1093/nar/gkv1367 26657629 10.1093/nar/gkv1367 PMC4756822

[23] Li P, Chen S, Chen H, Mo X, Li T, Shao Y, et al Using circular RNA as a novel type of biomarker in the screening of gastric cancer. Clinica chimica acta. 2015;444:132–6. 10.1016/j.cca.2015.02.018 25689795

[24] Bachmayr-Heyda A, Reiner AT, Auer K, Sukhbaatar N, Aust S, Bachleitner-Hofmann T, et al Correlation of circular RNA abundance with proliferation—exemplified with colorectal and ovarian cancer, idiopathic lung fibrosis, and normal human tissues. Scientific reports. 2015;5:8057 PubMed Central PMCID: PMC4306919. doi: 10.1038/srep08057 25624062 10.1038/srep08057 PMC4306919

[25] Bahn JH, Zhang Q, Li F, Chan TM, Lin X, Kim Y, et al The landscape of microRNA, Piwi-interacting RNA, and circular RNA in human saliva. Clinical chemistry. 2015;61(1):221–30. PubMed Central PMCID: PMC4332885. doi: 10.1373/clinchem.2014.230433 25376581 10.1373/clinchem.2014.230433 PMC4332885

[26] Lin X, Lo H-C, Wong DT, Xiao X. Noncoding RNAs in human saliva as potential disease biomarkers. Frontiers in genetics. 2015;6:175 doi: 10.3389/fgene.2015.00175 25999984 10.3389/fgene.2015.00175 PMC4423433

[27] Alhasan AA, Izuogu OG, Al-Balool HH, Steyn JS, Evans A, Colzani M, et al Circular RNA enrichment in platelets is a signature of transcriptome degradation. Blood. 2016;127(9):e1–e11. PubMed Central PMCID: PMC4797142. doi: 10.1182/blood-2015-06-649434 26660425 10.1182/blood-2015-06-649434 PMC4797142

[28] Memczak S, Papavasileiou P, Peters O, Rajewsky N. Identification and Characterization of Circular RNAs As a New Class of Putative Biomarkers in Human Blood. PloS ONE. 2015;10(10):e0141214 PubMed Central PMCID: PMC4617279. doi: 10.1371/journal.pone.0141214 26485708 10.1371/journal.pone.0141214 PMC4617279

[29] Koh W, Pan W, Gawad C, Fan HC, Kerchner GA, Wyss-Coray T, et al Noninvasive in vivo monitoring of tissue-specific global gene expression in humans. Proceedings of the National Academy of Sciences of the United States of America. 2014;111(20):7361–6. PubMed Central PMCID: PMC4034220. doi: 10.1073/pnas.1405528111 24799715 10.1073/pnas.1405528111 PMC4034220

[30] Li Y, Zheng Q, Bao C, Li S, Guo W, Zhao J, et al Circular RNA is enriched and stable in exosomes: a promising biomarker for cancer diagnosis. Cell research. 2015;25(8):981–4. PubMed Central PMCID: PMC4528056. doi: 10.1038/cr.2015.82 26138677 10.1038/cr.2015.82 PMC4528056

[31] Lasda E, Parker R. Circular RNAs Co-Precipitate with Extracellular Vesicles: A Possible Mechanism for circRNA Clearance. PloS ONE. 2016;11(2):e0148407 doi: 10.1371/journal.pone.0148407 26848835 10.1371/journal.pone.0148407 PMC4743949

[32] Guo JU, Agarwal V, Guo H, Bartel DP. Expanded identification and characterization of mammalian circular RNAs. Genome biology. 2014;15(7):409 PubMed Central PMCID: PMC4165365. doi: 10.1186/s13059-014-0409-z 25070500 10.1186/s13059-014-0409-z PMC4165365

[33] Szabo L, Morey R, Palpant NJ, Wang PL, Afari N, Jiang C, et al Statistically based splicing detection reveals neural enrichment and tissue-specific induction of circular RNA during human fetal development. Genome biology. 2015;16:126 PubMed Central PMCID: PMC4506483. doi: 10.1186/s13059-015-0690-5 26076956 10.1186/s13059-015-0690-5 PMC4506483

[34] Westholm JO, Miura P, Olson S, Shenker S, Joseph B, Sanfilippo P, et al Genome-wide analysis of drosophila circular RNAs reveals their structural and sequence properties and age-dependent neural accumulation. Cell reports. 2014;9(5):1966–80. PubMed Central PMCID: PMC4279448. doi: 10.1016/j.celrep.2014.10.062 25544350 10.1016/j.celrep.2014.10.062 PMC4279448

[35] Veno MT, Hansen TB, Veno ST, Clausen BH, Grebing M, Finsen B, et al Spatio-temporal regulation of circular RNA expression during porcine embryonic brain development. Genome biology. 2015;16:245 PubMed Central PMCID: PMC4635978. doi: 10.1186/s13059-015-0801-3 26541409 10.1186/s13059-015-0801-3 PMC4635978

[36] Salzman J, Chen RE, Olsen MN, Wang PL, Brown PO. Cell-type specific features of circular RNA expression. PLoS Genet. 2013;9(9):e1003777 PubMed Central PMCID: PMC3764148. doi: 10.1371/journal.pgen.1003777 24039610 10.1371/journal.pgen.1003777 PMC3764148

[37] Hansen TB, Veno MT, Damgaard CK, Kjems J. Comparison of circular RNA prediction tools. Nucleic acids research. 2016;44(6):e58 PubMed Central PMCID: PMC4824091. doi: 10.1093/nar/gkv1458 26657634 10.1093/nar/gkv1458 PMC4824091

[38] Song X, Zhang N, Han P, Moon BS, Lai RK, Wang K, et al Circular RNA profile in gliomas revealed by identification tool UROBORUS. Nucleic acids research. 2016;44(9):e87 PubMed Central PMCID: PMC4872085. doi: 10.1093/nar/gkw075 26873924 10.1093/nar/gkw075 PMC4872085

[39] Gao Y, Wang J, Zhao F. CIRI: an efficient and unbiased algorithm for de novo circular RNA identification. Genome biology. 2015;16:4 PubMed Central PMCID: PMC4316645. doi: 10.1186/s13059-014-0571-3 25583365 10.1186/s13059-014-0571-3 PMC4316645

[40] Chuang TJ, Wu CS, Chen CY, Hung LY, Chiang TW, Yang MY. NCLscan: accurate identification of non-co-linear transcripts (fusion, trans-splicing and circular RNA) with a good balance between sensitivity and precision. Nucleic acids research. 2016;44(3):e29 PubMed Central PMCID: PMC4756807. doi: 10.1093/nar/gkv1013 26442529 10.1093/nar/gkv1013 PMC4756807

[41] Izuogu OG, Alhasan AA, Alafghani HM, Santibanez-Koref M, Elliott DJ, Jackson MS. PTESFinder: a computational method to identify post-transcriptional exon shuffling (PTES) events. BMC bioinformatics. 2016;17:31 PubMed Central PMCID: PMC4711006. doi: 10.1186/s12859-016-0881-4 26758031 10.1186/s12859-016-0881-4 PMC4711006

[42] Cheng J, Metge F, Dieterich C. Specific identification and quantification of circular RNAs from sequencing data. Bioinformatics. 2016;32(7):1094–6. doi: 10.1093/bioinformatics/btv656 26556385 10.1093/bioinformatics/btv656

[43] Szabo L, Salzman J. Detecting circular RNAs: bioinformatic and experimental challenges. Nat Rev Genet. 2016;17(11):679–92. doi: 10.1038/nrg.2016.114 27739534 10.1038/nrg.2016.114 PMC5565156

[44] Glazar P, Papavasileiou P, Rajewsky N. circBase: a database for circular RNAs. Rna. 2014;20(11):1666–70. PubMed Central PMCID: PMC4201819. doi: 10.1261/rna.043687.113 25234927 10.1261/rna.043687.113 PMC4201819

[45] Guarnerio J, Bezzi M, Jeong JC, Paffenholz SV, Berry K, Naldini MM, et al Oncogenic Role of Fusion-circRNAs Derived from Cancer-Associated Chromosomal Translocations. Cell. 2016;165(2):289–302. doi: 10.1016/j.cell.2016.03.020 27040497 10.1016/j.cell.2016.03.020

[46] Li H. Aligning sequence reads, clone sequences and assembly contigs with BWA-MEM. arXiv preprint arXiv:13033997. 2013.

[47] Hoffmann S, Otto C, Doose G, Tanzer A, Langenberger D, Christ S, et al A multi-split mapping algorithm for circular RNA, splicing, trans-splicing and fusion detection. Genome biology. 2014;15(2):R34 PubMed Central PMCID: PMC4056463. doi: 10.1186/gb-2014-15-2-r34 24512684 10.1186/gb-2014-15-2-r34 PMC4056463

[48] Wang K, Singh D, Zeng Z, Coleman SJ, Huang Y, Savich GL, et al MapSplice: accurate mapping of RNA-seq reads for splice junction discovery. Nucleic acids research. 2010;38(18):e178 PubMed Central PMCID: PMC2952873. doi: 10.1093/nar/gkq622 20802226 10.1093/nar/gkq622 PMC2952873

[49] Dobin A, Davis CA, Schlesinger F, Drenkow J, Zaleski C, Jha S, et al STAR: ultrafast universal RNA-seq aligner. Bioinformatics. 2013;29(1):15–21. PubMed Central PMCID: PMC3530905. doi: 10.1093/bioinformatics/bts635 23104886 10.1093/bioinformatics/bts635 PMC3530905

[50] Trapnell C, Pachter L, Salzberg SL. TopHat: discovering splice junctions with RNA-Seq. Bioinformatics. 2009;25(9):1105–11. PubMed Central PMCID: PMC2672628. doi: 10.1093/bioinformatics/btp120 19289445 10.1093/bioinformatics/btp120 PMC2672628

[51] Kim D, Salzberg SL. TopHat-Fusion: an algorithm for discovery of novel fusion transcripts. Genome biology. 2011;12(8):R72 PubMed Central PMCID: PMC3245612. doi: 10.1186/gb-2011-12-8-r72 21835007 10.1186/gb-2011-12-8-r72 PMC3245612

[52] Langmead B, Salzberg SL. Fast gapped-read alignment with Bowtie 2. Nature methods. 2012;9(4):357–9. PubMed Central PMCID: PMC3322381. doi: 10.1038/nmeth.1923 22388286 10.1038/nmeth.1923 PMC3322381

[53] Li H, Durbin R. Fast and accurate short read alignment with Burrows-Wheeler transform. Bioinformatics. 2009;25(14):1754–60. PubMed Central PMCID: PMC2705234. doi: 10.1093/bioinformatics/btp324 19451168 10.1093/bioinformatics/btp324 PMC2705234

[54] Kent WJ. BLAT—the BLAST-like alignment tool. Genome research. 2002;12(4):656–64. PubMed Central PMCID: PMC187518. doi: 10.1101/gr.229202 11932250 10.1101/gr.229202 PMC187518

[55] Huang W, Li L, Myers JR, Marth GT. ART: a next-generation sequencing read simulator. Bioinformatics. 2012;28(4):593–4. PubMed Central PMCID: PMCPMC3278762. doi: 10.1093/bioinformatics/btr708 22199392 10.1093/bioinformatics/btr708 PMC3278762

